# Supplementary material for: Sex-stratified genome-wide association meta-analysis of major depressive disorder
Source: Nat Commun. 2025 Aug 26;16:7960. doi: 10.1038/s41467-025-63236-1 (PMC12381276; doi:10.1038/s41467-025-63236-1)
Supplement: Supplementary file 2 — Description of Additional Supplementary Files [file 41467_2025_63236_MOESM2_ESM.pdf]

Description of Additional Supplementary Files

Sex-stratified genome-wide association meta-analysis of Major Depressive Disorder

Jodi T. Thomas, Jackson G. Thorp, Floris Huider, Poppy Z. Grimes, Rujia Wang, Pierre Youssef, Jonathan R.I. Coleman, Enda M. Byrne, Mark Adams, BIONIC consortium, The GLAD Study, Sarah E. Medland, Ian B. Hickie, Catherine M. Olsen, David C. Whiteman, Heather C. Whalley, Brenda W.J.H. Penninx, Hanna M. van Loo, Eske M. Derks, Thalia C. Eley, Gerome Breen, Dorret I. Boomsma, Naomi R. Wray, Nicholas G. Martin, Brittany L. Mitchell

Contents

Supplementary Data 1 – 33

References from Additional Supplementary Files

Supplementary Data 1. **Cohorts included in the meta-analyses.** ASR-ASEBA = Adult Self-report - The Achenbach System of Empirically Based Assessment. BDI = Beck's Depression Inventory. CES-D = Center for Epidemiologic Studies Depression Scale. CIDI-SF = Composite International Diagnostic Interview short form. DSM = Diagnostic and Statistical Manual of Mental Disorders. EHR = electronic health records. GxS = genome-wide genotype-by-sex interaction analysis. HADS = Hospital Anxiety and Depression Scale. ICD-10 = International Classification of Diseases-10. LIDAS = Lifetime Depression Assessment Survey. MDD = major depressive disorder. MINI = Mini-International Neuropsychiatric Interview. PGC = Psychiatric Genomics Consortium. iPSYCH = The Integrative Psychiatric Research consortium which has established a large Danish population-based Case–Cohort sample.

Supplementary Data 2. **Traits that have previously been found to be associated with each of the genome-wide significant independent lead SNPs, or any SNPs in linkage disequilibrium with these independent SNPs, in our female GWAS.** Associated traits were determined by the list of SNPs in GWASCatalog. As the largest GWAS of major depressive disorder was not yet published when doing this analysis all significant SNPs, and any SNPs in linkage disequilibrium, from Adams *et al.*, [1] were also searched and have been added as the Trait 'Adams Major Depressive Disorder'.

Supplementary Data 3. **Traits that have previously been found to be associated with each of the genome-wide significant independent lead SNPs, or any SNPs in linkage disequilibrium with these independent SNPs, in our male GWAS.** Associated traits were determined by the list of SNPs in GWASCatalog. As the largest GWAS of major depressive disorder was not yet published when doing this analysis all significant SNPs, and any SNPs in linkage disequilibrium, from Adams *et al.*, [1] were also searched and have been added as the Trait 'Adams Major Depressive Disorder'. Bolded is the independent lead SNP for which no depression traits have previously been associated with this SNP or any SNPs in linkage disequilibrium with it.

Supplementary Data 4. **Genetic correlation results between our sex-stratified GWAS meta-analysis of Major Depressive Disorder (MDD) and previously published GWAS of depression.** Genetic correlation was estimated using linkage disequilibrium score regression (LDSC) between our sex-stratified GWAS meta-analysis of MDD (Females: 130,471 cases, 159,521 controls. Males: 64,805 cases, 132,185 controls) with the largest published GWAS meta-analysis of MDD in both sexes combined (Adams *et al.,* [1]) and with previously published sex-stratified GWAS of MDD (Blokland *et al.,* [2]; Silveira *et al.,* [3]). The two-sided p-value tests whether the genetic correlation is significantly different from zero and was adjusted for 10 comparisons using the Benjamini-Hochberg method. SE = standard error. Z = Z statistic. P = P-value. Adjusted P = P-value adjusted for 10 comparisons.

Supplementary Data 5. **Comparison between the genetic correlation of our Major Depressive Disorder (MDD) GWAS in females with the previously published MDD GWAS versus the genetic correlation of our MDD GWAS in males with the same previously published MDD GWAS.** Genetic correlations were estimated using linkage disequilibrium score regression (LDSC) using our sex-stratified GWAS summary statistics (Females: 130,471 cases, 159,521 controls. Males: 64,805 cases, 132,185 controls) and publicly available GWAS datasets for depression; the largest published GWAS for MDD (sex-combined) (Adams *et al.,* [1]) and previous sex-stratified GWAS for depression (Blokland *et al.,* [2] and Silveira *et al.,* [3]). Assessed using the jack-knife method and a two-sided Z-test on the difference in genetic correlations across the 200 jack-knife pseudo-values. P-values were adjusted using the Benjamini Hochberg method for five tests. The results from the Z-score method using the two-sided Z-test are also presented as it is commonly used, but not theoretically appropriate. Z = Z-score, a positive Z-score indicates that the genetic correlation between our female MDD vs previous MDD is larger than the genetic correlation between our male MDD vs previous MDD. P = P-value. Adjusted P = P-value adjusted for five comparisons.

Supplementary Data 6. **Comparison of the results from our female GWAS meta-analysis to the replication in the Generation Scotland cohort using females only, for the 16 independent genome-wide significant SNPs identified in our female GWASmeta-analysis.** Our female GWAS meta-analysis: 130,471 cases and 159,521 controls. The

Generation Scotland replication GWAS in females: 2,441 cases and 3,321 controls. rsID = SNP ID, CHR = chromosome, BP = base pair location (according to human genome build 37), Allele1 = effect/tested allele, Allele2 = alternative allele, Effect = effect size (beta), SE = standard error of effect size, P = p-value of effect in the GWAS (not adjusted for multiple comparisons), FREQA1 = Frequency of allele1, Effect_same_direction = is the effect size in the same direction for the female GWAS meta-analysis and the replication in Generation Scotland using females only, Meta = our female GWAS meta-analysis results, GenScot = replication results in Generation Scotland cohort, Effect_Meta_minus_GenScot = effect size in our female GWAS meta-analysis minus the effect size in the Generation Scotland replication.

Supplementary Data 7. **Comparison of the results from our male GWAS meta-analysis to the replication in the Generation Scotland cohort using males only.** We identified eight independent genome-wide significant SNPs in our male GWAS meta-analysis, but only seven SNPs were tested for replication as one SNP was unavailable in the Generation Scotland cohort. Our male GWAS meta-analysis: 64,805 cases and 132,185 controls. The Generation Scotland replication GWAS in males: 938 cases and 2,491 controls. rsID = SNP ID, CHR = chromosome, BP = base pair location (according to human genome build 37), Allele1 = effect/tested allele, Allele2 = alternative allele, Effect = effect size (beta), SE = standard error of effect size, P = p-value of effect in the GWAS (not adjusted for multiple comparisons), FREQA1 = Frequency of allele1, Effect_same_direction = is the effect size in the same direction for the male GWAS meta-analysis and the replication in Generation Scotland using males only, Meta = our male GWAS meta-analysis results, GenScot = replication results in Generation Scotland cohort, Effect_Meta_minus_GenScot = effect size in our male GWAS meta-analysis minus the effect size in the Generation Scotland replication.

Supplementary Data 8. **Univariate MiXeR results quantifying polygenicity using our sex-stratified GWAS meta-analysis results.** MiXeR fits a Gaussian mixture model assuming that common genetic effects on a trait are a mixture of causal variants and non-causal variants. Females: 130,471 cases, 159,521 controls. Males: 64,805 cases, 132,185 controls. pi = polygenicity; sig2_beta = discoverability; sig2_zero = chi-square statistic inflation factor;

h2 = heritability; nc@p9 = number of causal variants explaining 90% of heritability; AIC = Akaike information criterion; BIC = Bayesian information criterion; MDD = major depressive disorder.

Supplementary Data 9. **Univariate MiXeR results quantifying polygenicity in females and males of the UK Biobank full and down-sampled cohort.** MiXeR fits a Gaussian mixture model assuming that common genetic effects on a trait are a mixture of causal variants and non-causal variants. The full UK Biobank sample: Females = 46,194 cases and 53,211 controls, Males = 22,608 cases and 56,516 controls. UK Biobank after down-sampling: n = 22,608 cases and 53,211 in both females and males. pi = polygenicity; sig2_beta = discoverability; sig2_zero = chi-square statistic inflation factor; h2 = heritability; nc@p9 = number of causal variants explaining 90% of heritability; AIC = Akaike information criterion; BIC = Bayesian information criterion; MDD = major depressive disorder

Supplementary Data 10. **The posterior probability that autosomal SNP-based heritability (h2) on the liability scale is higher in females compared to males.** SNP-based heritability was estimated using SBayesS with our sex-stratified GWAS meta-analysis results (Females: 130,471 cases, 159,521 controls. Males: 64,805 cases, 132,185 controls). When estimating SNP-based heritability for males, the proportion of unscreened controls ranged from 0 – 1 (i.e. all controls screened – no screening of controls) and the corresponding population prevalence ranged from 0.1 - 0.2. In females, SNP-based heritability was estimated using a population prevalence of 0.2 and no unscreened controls. Bolded are the scenarios in which there is at least moderate evidence (>80% posterior probability) that female h2 > male h2. H2 = autosomal SNP-based heritability.

Supplementary Data 11. **Bivariate MiXeR results examining polygenic overlap between our GWAS meta-analyses of Major Depressive Disorder (MDD) in females and males.** Females: 130,471 cases, 159,521 controls. Males: 64,805 cases, 132,185 controls. nc1, nc2, and nc12 = number of causal variants explaining 90% of heritability that are specific to trait 1, specific to trait 2, and shared between trait 1 and 2, respectively; rho = correlation of effect sizes in shared polygenic component; rg = genetic correlation.

Supplementary Data 12. **Bivariate MiXeR results investigating polygenic overlap between Major Depressive Disorder (MDD) in females and males using the full and down-sampled UK Biobank cohort.** The full UK Biobank sample: Females = 46,194 cases and 53,211 controls, Males = 22,608 cases and 56,516 controls. UK Biobank after down-sampling: n = 22,608 cases and 53,211 in both females and males. nc1, nc2, and nc12 = number of causal variants explaining 90% of heritability that are specific to trait 1, specific to trait 2, and shared between trait 1 and 2, respectively; rho = correlation of effect sizes in shared polygenic component; rg = genetic correlation.

Supplementary Data 13. **Information and possible causal variants for the 42 genomic regions that share a common causal variant between Major Depressive Disorder (MDD) in females and MDD in males as identified by gwas-pw.** Of these 42 shared regions, four variants (in bold) were identified as possible causal risk loci (PPA of model 3 (shared model) > 0.5). For each genomic region, the regions position (base pair position according to human genome build 37) and posterior probability that this region is shared by MDD in females and males is displayed. Information for the SNP with the highest posterior probability of being the causal SNP in each region is also displayed: rsID, base pair position according to human genome build 37 (SNP pos), effect allele and other allele. For each variant with a posterior probability of being the causal variant > 0.5, i.e. for each possible causal risk variant, the effect size (beta), standard error (SE), two-sided unadjusted p-value (P) and frequency of the effect allele from the GWAS results in females and males are displayed. Gene nearest SNP = The gene nearest the possible causal SNP (with the distance and function in brackets). Genes (annotated with > 1 method) = All genes annotated to the possible causal SNP with more than 1 method (from positional, eQTL and chromatin interaction mapping). Gene: poisitional = the genes mapped to each of the possible causal SNPs using positional mapping. Gene: eQTL = the genes mapped to each of the possible causal SNPs using eQTL information, with the tissue type in brackets next to each gene. Gene: chromatin interaction = the genes mapped to each of the possible causal SNPs using chromatin interaction information, with the tissue type in brackets next to each gene.

Supplementary Data 14. **Information for the three genomic regions that contain a causal variant for Major Depressive Disorder (MDD) in females only as identified by gwas-pw.** For each genomic region, the regions position (base pair position according to human genome build 37) and posterior probability that this region is specific to MDD in females is displayed. Information for the SNP with the highest posterior probability of being the causal SNP in each region is also displayed: rsID, base pair position according to human genome build 37 (SNP pos), effect allele and other allele. None of these female-specific MDD regions contained a SNP with a posterior probability of being the causal variant > 0.5, i.e. no SNPs were identified as possible causal risk loci for MDD in females only.

Supplementary Data 15. **Independent lead SNPs identified as genome-wide significant in the GWAS meta-analysis of Major Depressive Disorder (MDD) in females.** rsID = SNP ID, Chr = chromosome, Pos = base pair location (according to human genome build 37). For each SNP, results from the female GWAS, male GWAS and GxS analysis are displayed: the effect size (Beta), standard error (SE), two-sided unadjusted p-value (P) and frequency of the effect allele from the GWAS results (Freq Effect Allele). Significant SNPs in LD = other SNPs identified as genome-wide significant in the female GWAS that are in linkage disequilibrium with each of the independent lead SNPs. Gene nearest lead SNP = The gene nearest the lead SNP (with the distance and function in brackets). Gene nearest significant SNPs in LD = The gene nearest each of the SNPs identified as genome-wide significant in the female GWAS that are in linkage disequilibrium with each of the independent lead SNPs (the distance and function in brackets). Genes (annotated with > 1 method) = All genes annotated to the lead SNP, and any genome-wide significant SNPs in linkage disequilibrium with the lead SNP, with more than one method (from positional, eQTL and chromatin interaction mapping).Gene: poisitional = the genes mapped to each of the independent lead SNPs (or any of the genome-wide significant SNPs in LD with these lead SNPs) using positional mapping. Gene: eQTL = the genes mapped to each of the independent lead SNPs (or any of the genome-wide significant SNPs in LD with these lead SNPs) using eQTL information, with the tissue type in brackets next to each gene. Gene: chromatin interaction = the genes mapped to each of the independent lead SNPs (or any of the genome-wide significant SNPs in LD with these lead SNPs) using chromatin interaction information, with the tissue type in brackets next to each gene.

Supplementary Data 16. **Independent lead SNPs identified as genome-wide significant in the GWAS meta-analysis of Major Depressive Disorder (MDD) in males.** rsID = SNP ID, Chr = chromosome, Pos = base pair location (according to human genome build 37). For each SNP, results from the male GWAS, female GWAS and GxS analysis are displayed: the effect size (Beta), standard error (SE), two-sided unadjusted p-value (P) and frequency of the effect allele from the GWAS results (Freq Effect Allele). Significant SNPs in LD = other SNPs identified as genome-wide significant in the male GWAS that are in linkage disequilibrium with each of the independent lead SNPs. Gene nearest lead SNP = The gene nearest the lead SNP (with the distance and function in brackets). Gene nearest significant SNPs in LD = The gene nearest each of the SNPs identified as genome-wide significant in the male GWAS that are in linkage disequilibrium with each of the independent lead SNPs (the distance and function in brackets). Genes (annotated with > 1 method) = All genes annotated to the lead SNP, and any genome-wide significant SNPs in linkage disequilibrium with the lead SNP, with more than one method (from positional, eQTL and chromatin interaction mapping). Gene: poisitional = the genes mapped to each of the independent lead SNPs (or any of the genome-wide significant SNPs in LD with these lead SNPs) using positional mapping. Gene: eQTL = the genes mapped to each of the independent lead SNPs (or any of the genome-wide significant SNPs in LD with these lead SNPs) using eQTL information, with the tissue type in brackets next to each gene. Gene: chromatin interaction = the genes mapped to each of the independent lead SNPs (or any of the genome-wide significant SNPs in LD with these lead SNPs) using chromatin interaction information, with the tissue type in brackets next to each gene.

Supplementary Data 17. **A comparison across sexes of the genes annotated to the genome-wide significant SNPs identified in the sex-stratified GWAS meta-analysis of Major Depressive Disorder (MDD).** All independent, lead, genome-wide significant SNPs, and any genome-wide significant SNPs in linkage disequilibrium with them, were mapped to genes with positional, eQTL and chromatin interaction mapping. All genes annotated to a SNP with more than one of these mapping methods were retained. These genes were compared across females and males. Only one gene was found in both sexes (bolded). cross = gene not

mapped to the genome-wide significant SNPs in this sex, tick = gene is mapped to the genome-wide significant SNPs in this sex.

Supplementary Data 18. **Comparison of the direction of association between our one novel Major Depressive Disorder (MDD) SNP and this SNP's association with other traits.** Our sex-stratified GWAS meta-analysis of MDD identified one novel SNP (rs5971319) which has not previously been associated with any depression phenotypes. This novel SNP is on the X chromosome and was identified in our male GWAS. Here, the direction of association of this SNP with other traits is displayed, as identified in GWAS Catalog. SNP = rsID of our novel variant (rs5971319). SNP in LD = rsID of the SNP in linkage disequilibrium with rs5971319. Trait = Other trait which this SNP in linkage disequilibrium has previously been associated with. Tested Allele male GWAS meta-analysis = the tested allele in our GWAS meta-analysis of MDD in males. Tested Allele in other trait = the tested allele in the GWAS results for the trait. Beta of SNP in LD in male GWAS meta-analysis = Effect size (beta) of the SNP in linkage disequilibrium with rs5971319 in our GWAS meta-analysis of MDD in males. Beta of SNP in LD in trait = Effect size (beta) of the SNP in linkage disequilibrium with rs5971319 in the GWAS of the other trait. Effect direction for MDD vs other trait = based on the tested alleles and effect sizes, whether the association between the SNP and MDD and between the SNP and the other trait is in the same or opposite directions.

Supplementary Data 19. **Traits that have previously been found to be associated with each of the nominally significant independent lead SNPs, or any SNPs in linkage disequilibrium with these independent SNPs, in our genotype-by-sex interaction analysis.** Associated traits were determined by the list of SNPs in GWAS Catalog. As the largest GWAS of major depressive disorder (Adams *et al.,* [1]) was not yet published when doing this analysis all significant SNPs, and any SNPs in linkage disequilibrium, from Adams *et al.,* [1] were also searched and have been added as the Trait 'Adams Major Depressive Disorder'. Bolded are all four independent lead SNPs for which no depression traits have previously been associated with this SNP or any SNPs in linkage disequilibrium with it.

Supplementary Data 20. **Independent lead SNPs identified as nominally significant in the genome-wide genotype-by-sex (GxS) interaction meta-analysis of Major Depressive Disorder (MDD).** rsID = SNP ID, Chr = chromosome, Pos = base pair location (according to human genome build 37). For each SNP, results from the GxS analysis, female GWAS and male GWAS are displayed: the effect size (Beta), standard error (SE), two-sided unadjusted p-value (P) and frequency of the effect allele from the results (Freq Effect Allele). Significant SNPs in LD = other SNPs identified as nominally significant in the GxS analysis that are in linkage disequilibrium with each of the independent lead SNPs. Gene nearest lead SNP = The gene nearest the lead SNP (with the distance and function in brackets). Gene nearest significant SNPs in LD = The gene nearest each of the SNPs identified as nominally significant in the GxS analysis that are in linkage disequilibrium with each of the independent lead SNPs (the distance and function in brackets). Genes (annotated with > 1 method) = All genes annotated to the lead SNP, and any nominally significant SNPs in linkage disequilibrium with the lead SNP, with more than one method (from positional, eQTL and chromatin interaction mapping). Gene: poisitional = the genes mapped to each of the independent lead SNPs (or any of the nominally significant SNPs in LD with these lead SNPs) using positional mapping. Gene: eQTL = the genes mapped to each of the independent lead SNPs (or any of the nominally significant SNPs in LD with these lead SNPs) using eQTL information, with the tissue type in brackets next to each gene. Gene: chromatin interaction = the genes mapped to each of the independent lead SNPs (or any of the nominally significant SNPs in LD with these lead SNPs) using chromatin interaction information, with the tissue type in brackets next to each gene.

Supplementary Data 21. **Traits included in linkage disequilibrium score (LDSC) regression to estimate genome-wide autosomal SNP-based genetic correlation with our sex-stratified MDD GWAS meta-analysis results.**

Supplementary Data 22**. Genetic correlation results between our sex-stratified GWAS meta-analysis of Major Depressive Disorder (MDD) and a range of traits.** Genetic correlation was estimated using linkage disequilibrium score regression (LDSC) between our sex-stratified GWAS meta-analysis of MDD (Females: 130,471 cases, 159,521 controls. Males: 64,805 cases, 132,185 controls) with previous traits, as outlined in Supplementary

Data 21. The two-sided p-value tests whether the genetic correlation is significantly different from zero and was adjusted for 22 comparisons using the Benjamini-Hochberg method. SE = standard error. Z = Z statistic. P = P-value. Adjusted P = P-value adjusted for 22 comparisons.

Supplementary Data 23. **Comparison between the genetic correlation of our Major Depressive Disorder (MDD) GWAS in females with the GWAS of another trait versus the genetic correlation of our MDD GWAS in males with the same trait.** Genetic correlations were estimated using linkage disequilibrium score regression (LDSC) using our sex-stratified GWAS summary statistics (Females: 130,471 cases, 159,521 controls. Males: 64,805 cases, 132,185 controls) and publicly available GWAS datasets for other traits (Supplementary Data 21). Assessed using the jack-knife method and a two-sided Z-test on the difference in genetic correlations across the 200 jack-knife pseudo-values. P-values were adjusted using the Benjamini Hochberg method for 11 tests. The results from the Z-score method using the two-sided Z-test are also presented as it is commonly used, but not theoretically appropriate. Z = Z-score, a positive Z-score indicates that the genetic correlation between our female MDD vs other trait is larger than the genetic correlation between our male MDD vs other trait. P = P-value. Adjusted P = P-value adjusted for 11 comparisons.

Supplementary Data 24. **Genetic correlation results between our sex-stratified GWAS meta-analysis of Major Depressive Disorder (MDD) and sex-stratified body mass index (BMI).** Genetic correlation was estimated using linkage disequilibrium score regression (LDSC) between our sex-stratified GWAS meta-analysis of MDD (Females: 130,471 cases, 159,521 controls. Males: 64,805 cases, 132,185 controls) with sex-stratified BMI, as outlined in Supplementary Data 21. The two-sided p-value tests whether the genetic correlation is significantly different from zero and was adjusted for four comparisons using the Benjamini-Hochberg method. SE = standard error. Z = Z statistic. P = P-value. Adjusted P = P-value adjusted for four comparisons.

Supplementary Data 25. **Comparison between the genetic correlation of our Major Depressive Disorder (MDD) GWAS in females with the body mass index (BMI) GWAS**

in females versus the genetic correlation of our MDD GWAS in males with the BMI GWAS in males. Genetic correlations were estimated using linkage disequilibrium score regression (LDSC) using our sex-stratified GWAS summary statistics (Females: 130,471 cases, 159,521 controls. Males: 64,805 cases, 132,185 controls) and the publicly available sex-stratified GWAS datasets of BMI (Pulit *et al.,* [4]). Assessed using the jack-knife method and a two-sided Z-test on the difference in genetic correlations across the 200 jack-knife pseudo-values. P-values were adjusted using the Benjamini Hochberg method for two tests. The results from the Z-score method using the two-sided Z-test are also presented as it is commonly used, but not theoretically appropriate. Z = Z-score, a positive Z-score indicates that the genetic correlation between our female MDD vs female BMI is larger than the genetic correlation between our male MDD vs male BMI. P = P-value. Adjusted P = P-value adjusted for two comparisons.

Supplementary Data 26. **Univariate MiXeR results quantifying polygenicity for sex-stratified body mass index (BMI) and sex-combined metabolic syndrome (metS).** MiXeR fits a Gaussian mixture model assuming that common genetic effects on a trait are a mixture of causal variants and non-causal variants. pi = polygenicity; sig2_beta = discoverability; sig2_zero = chi-square statistic inflation factor; h2 = heritability; nc@p9 = number of causal variants explaining 90% of heritability; AIC = Akaike information criterion; BIC = Bayesian information criterion.

Supplementary Data 27. **Bivariate MiXeR results examining polygenic overlap between our GWAS meta-analyses of Major Depressive Disorder (MDD) in females/males and the metabolic traits body mass index (BMI) (sex-stratified) and metabolic syndrome (metS) (sex-combined).** Our sex-stratified GWAS meta-analysis of MDD = Females: 130,471 cases, 159,521 controls. Males: 64,805 cases, 132,185 controls. nc1, nc2, and nc12 = number of causal variants explaining 90% of heritability that are specific to trait 1, specific to trait 2, and shared between trait 1 and 2, respectively; rho = correlation of effect sizes in shared polygenic component; rg = genetic correlation.

Supplementary Data 28. **Information and possible causal variants for the one genomic region that shares a common causal variant between Major Depressive Disorder (MDD) and body mass index (BMI) in both sexes, as identified by gwas-pw.** For this genomic region shared by MDD and BMI, the regions position (base pair position according to human genome build 37) and posterior probability that this region is shared by MDD and BMI is displayed. Information for the SNP with the highest posterior probability of being the causal SNP in this region for females and males is also displayed: rsID, base pair position according to human genome build 37 (SNP pos), effect allele and other allele. In this one shared region, a different variant was identified as a possible causal risk locus (PPA of model 3 (shared model) > 0.5) in females and males. Thus, two rows are displayed for the same genomic region: one with the possible causal variant identified in females, and one with the possible causal variant identified in males. As each variant has a posterior probability of being the causal variant > 0.5, i.e. they are both possible causal risk variants, the effect size (beta), standard error (SE), two-sided unadjusted p-value (P) and frequency of the effect allele from the GWAS results for MDD in females and males and BMI in females and males are displayed. Gene nearest SNP = The gene nearest the possible causal SNP (with the distance and function in brackets). Genes (annotated with > 1 method) = All genes annotated to the possible causal SNP with more than 1 method (from positional, eQTL and chromatin interaction mapping). Gene: poisitional = the genes mapped to each of the possible causal SNPs using positional mapping. Gene: eQTL = the genes mapped to each of the possible causal SNPs using eQTL information, with the tissue type in brackets next to each gene. Gene: chromatin interaction = the genes mapped to each of the possible causal SNPs using chromatin interaction information, with the tissue type in brackets next to each gene. Note that the two different possible causal risk variants identified in females and males from the same genomic region shared by MDD and BMI map to the same genes.

Supplementary Data 29. **Information and possible causal variants for the 24 genomic region that share a common causal variant between Major Depressive Disorder (MDD) and body mass index (BMI) in females only, as identified by gwas-pw.** Of these 24 shared regions, six variants (in bold) were identified as possible causal risk loci (PPA of model 3 (shared model) > 0.5). For each genomic region, the regions position (base pair position according to human genome build 37) and posterior probability that this region is shared by MDD in females and BMI in females is displayed. Information for the SNP with the highest

posterior probability of being the causal SNP in each region is also displayed: rsID, base pair position according to human genome build 37 (SNP pos), effect allele and other allele. For each variant with a posterior probability of being the causal variant > 0.5, i.e. for each possible causal risk variant, the effect size (beta), standard error (SE), two-sided unadjusted p-value (P) and frequency of the effect allele from the GWAS results for MDD in females and BMI in females are displayed. Gene nearest SNP = The gene nearest the possible causal SNP (with the distance and function in brackets). Genes (annotated with > 1 method) = All genes annotated to the possible causal SNP with more than 1 method (from positional, eQTL and chromatin interaction mapping). Gene: poisitional = the genes mapped to each of the possible causal SNPs using positional mapping. Gene: eQTL = the genes mapped to each of the possible causal SNPs using eQTL information, with the tissue type in brackets next to each gene. Gene: chromatin interaction = the genes mapped to each of the possible causal SNPs using chromatin interaction information, with the tissue type in brackets next to each gene.

Supplementary Data 30. **Information and possible causal variants for the four genomic regions that share a common causal variant between Major Depressive Disorder (MDD) and metabolic syndrome (metS) in both sexes, as identified by gwas-pw.** For these four genomic regions shared by MDD and metS, the regions position (base pair position according to human genome build 37) and posterior probability that this region is shared by MDD and metS is displayed. Information for the SNP with the highest posterior probability of being the causal SNP in this region for females and males is also displayed: rsID, base pair position according to human genome build 37 (SNP pos), effect allele and other allele (two rows for each genomic region - one with female top SNP and one with male top SNP). Of the four MDD/metS pleiotropic regions found in both sexes, four variants (shown in bold) were identified as possible causal risk loci (PPA of model 3 (shared model) > 0.5) in at least one sex. Only one possible causal variant was identified as the same in both sexes (yellow highlight). For possible risk variants, the effect size (beta), standard error (SE), two-sided unadjusted p-value (P) and frequency of the effect allele from the GWAS results for MDD in females and males and sex-combined metS are displayed. Gene nearest SNP = The gene nearest the possible causal SNP (with the distance and function in brackets). Genes (annotated with > 1 method) = All genes annotated to the possible causal SNP with more than 1 method (from positional, eQTL and chromatin interaction mapping). Gene: poisitional =

the genes mapped to each of the possible causal SNPs using positional mapping. Gene: eQTL = the genes mapped to each of the possible causal SNPs using eQTL information, with the tissue type in brackets next to each gene. Gene: chromatin interaction = the genes mapped to each of the possible causal SNPs using chromatin interaction information, with the tissue type in brackets next to each gene.

Supplementary Data 31. **Information and possible causal variants for the 22 genomic region that share a common causal variant between Major Depressive Disorder (MDD) and metabolic syndrome (metS) in females only, as identified by gwas-pw.** Of these 22 shared regions, five variants (in bold) were identified as possible causal risk loci (PPA of model 3 (shared model) > 0.5). For each genomic region, the regions position (base pair position according to human genome build 37) and posterior probability that this region is shared by MDD in females and sex-combined metS is displayed. Information for the SNP with the highest posterior probability of being the causal SNP in each region is also displayed: rsID, base pair position according to human genome build 37 (SNP pos), effect allele and other allele. For each variant with a posterior probability of being the causal variant > 0.5, i.e. for each possible causal risk variant, the effect size (beta), standard error (SE), two-sided unadjusted p-value (P) and frequency of the effect allele from the GWAS results for MDD in females and sex-combined metS are displayed. Gene nearest SNP = The gene nearest the possible causal SNP (with the distance and function in brackets). Genes (annotated with > 1 method) = All genes annotated to the possible causal SNP with more than 1 method (from positional, eQTL and chromatin interaction mapping). Gene: poisitional = the genes mapped to each of the possible causal SNPs using positional mapping. Gene: eQTL = the genes mapped to each of the possible causal SNPs using eQTL information, with the tissue type in brackets next to each gene. Gene: chromatin interaction = the genes mapped to each of the possible causal SNPs using chromatin interaction information, with the tissue type in brackets next to each gene.

Supplementary Data 32. **Information and possible causal variants for the four genomic region that share a common causal variant between Major Depressive Disorder (MDD) and metabolic syndrome (metS) in males only, as identified by gwas-pw.** Of these four shared regions, one variant (in bold) was identified as a possible causal risk locus (PPA of

model 3 (shared model) > 0.5). For each genomic region, the regions position (base pair position according to human genome build 37) and posterior probability that this region is shared by MDD in males and sex-combined metS is displayed. Information for the SNP with the highest posterior probability of being the causal SNP in each region is also displayed: rsID, base pair position according to human genome build 37 (SNP pos), effect allele and other allele. For the variant with a posterior probability of being the causal variant > 0.5, i.e. for the possible causal risk variant, the effect size (beta), standard error (SE), two-sided unadjusted p-value (P) and frequency of the effect allele from the GWAS results for MDD in males and sex-combined metS is displayed. Gene nearest SNP = The gene nearest the possible causal SNP (with the distance and function in brackets). Genes (annotated with > 1 method) = All genes annotated to the possible causal SNP with more than 1 method (from positional, eQTL and chromatin interaction mapping). Gene: poisitional = the genes mapped to each of the possible causal SNPs using positional mapping. Gene: eQTL = the genes mapped to each of the possible causal SNPs using eQTL information, with the tissue type in brackets next to each gene. Gene: chromatin interaction = the genes mapped to each of the possible causal SNPs using chromatin interaction information, with the tissue type in brackets next to each gene.

Supplementary Data 33. **Summary of how the recommended best practices for sex-aware analyses by Khramtsova *et al.*, [5] were addressed.**

References from Additional Supplementary Files

1. Adams MJ, et al., *Trans-ancestry genome-wide study of depression identifies 697 associations implicating cell types and pharmacotherapies.* Cell, 2025. **188**:1-13.

2. Blokland GAM, et al., *Sex-Dependent Shared and Nonshared Genetic Architecture Across Mood and Psychotic Disorders.* Biol Psychiatry, 2022. **91**(1):102-117.

3. Silveira PP, et al., *A sex-specific genome-wide association study of depression phenotypes in UK Biobank.* Mol Psychiatry, 2023. **28**:2469–2479.

4. Pulit SL, et al., *Meta-analysis of genome-wide association studies for body fat distribution in 694 649 individuals of European ancestry.* Hum Mol Genet, 2018. **28**(1):166-174.

5. Khramtsova EA, et al., *Quality control and analytic best practices for testing genetic models of sex differences in large populations.* Cell, 2023. **186**(10):2044-2061.
