# Supplementary material for: Sex-stratified genome-wide association meta-analysis of major depressive disorder
Source: Nat Commun. 2025 Aug 26;16:7960. doi: 10.1038/s41467-025-63236-1 (PMC12381276; doi:10.1038/s41467-025-63236-1)
Supplement: Supplementary file 4 — Reporting Summary [file 41467_2025_63236_MOESM4_ESM.pdf]

nature portfolio

Corresponding author(s): Jodi Thea Thomas

Last updated by author(s): Aug 7, 2025

Reporting Summary

Nature Portfolio wishes to improve the reproducibility of the work that we publish. This form provides structure for consistency and transparency in reporting. For further information on Nature Portfolio policies, see our Editorial Policies and the Editorial Policy Checklist.

Statistics

For all statistical analyses, confirm that the following items are present in the figure legend, table legend, main text, or Methods section.

n/a	Confirmed	
☐	☒	The exact sample size (*n*) for each experimental group/condition, given as a discrete number and unit of measurement
☐	☒	A statement on whether measurements were taken from distinct samples or whether the same sample was measured repeatedly
☐	☒	The statistical test(s) used AND whether they are one- or two-sided<br>*Only common tests should be described solely by name; describe more complex techniques in the Methods section.*
☐	☒	A description of all covariates tested
☐	☒	A description of any assumptions or corrections, such as tests of normality and adjustment for multiple comparisons
☐	☒	A full description of the statistical parameters including central tendency (e.g. means) or other basic estimates (e.g. regression coefficient) AND variation (e.g. standard deviation) or associated estimates of uncertainty (e.g. confidence intervals)
☐	☒	For null hypothesis testing, the test statistic (e.g. *F*, *t*, *r*) with confidence intervals, effect sizes, degrees of freedom and *P* value noted<br>*Give P values as exact values whenever suitable.*
☐	☒	For Bayesian analysis, information on the choice of priors and Markov chain Monte Carlo settings
☒	☐	For hierarchical and complex designs, identification of the appropriate level for tests and full reporting of outcomes
☐	☒	Estimates of effect sizes (e.g. Cohen's *d*, Pearson's *r*), indicating how they were calculated

Our web collection on statistics for biologists contains articles on many of the points above.

Software and code

Policy information about availability of computer code

Data collection

Data used in this publication had previously been collected as described in the published cohort profiles.

Australian Genetics of Depression Study (AGDS) + QSkin Sun and Health Study (QSkin):
Byrne EM, et al., Cohort profile: the Australian genetics of depression study. BMJ Open, 2020. 10(5):e032580.
Olsen CM, et al., Cohort profile: The QSkin Sun and Health Study. Int J Epidemiol, 2012. 41(4):929-929i.

The BIObanks Netherlands Internet Collaboration (BIONIC):
Huider F, et al., Genomics Research of Lifetime Depression in the Netherlands: The BIObanks Netherlands Internet Collaboration (BIONIC) Project. Twin Research and Human Genetics, 2024. 27(1):1-11.

GLAD+: The GLAD+ Study combines two United Kingdom (UK) cohorts: the Genetic Links to Anxiety and Depression (GLAD) Study (www.gladstudy.org.uk), and the National Institute for Health and Care Research (NIHR) BioResource COVID-19 Psychiatry and Neurological Genetics (COPING) Study.
Davies MR, et al., The Genetic Links to Anxiety and Depression (GLAD) Study: Online recruitment into the largest recontactable study of depression and anxiety. Behaviour Research and Therapy, 2019. 123:103503.

Generation Scotland:
Smith BH, et al., Cohort Profile: Generation Scotland: Scottish Family Health Study (GS:SFHS). The study, its participants and their potential for genetic research on health and illness. Int J Epidemiol, 2013. 42(3):689-700.

UK Biobank:
Bycroft C, et al., The UK Biobank resource with deep phenotyping and genomic data. Nature, 2018. 562(7726):203-209.

All Of Us:
All of Us Research Program Investigators, The “All of Us” research program. New England Journal of Medicine, 2019. 381(7):668-676.

Data analysis

Software used in data analyses:
GCTA v1.94.1
METAL (released 05/05/2020)
PLINK v1.90b6.8
GCTB v2.5.2
Linkage Disequilibrium Score Regression (LDSC) (released 13/02/2015)
R v4.3.1
MiXeR v1.3
gwas-pw v0.21
fgwas v0.3.6
SNP2GENE from FUMA v1.5.2
MAGMA v1.08 within the SNP2GENE function in FUMA v1.5.2

For manuscripts utilizing custom algorithms or software that are central to the research but not yet described in published literature, software must be made available to editors and reviewers. We strongly encourage code deposition in a community repository (e.g. GitHub). See the Nature Portfolio guidelines for submitting code & software for further information.

Data

Policy information about availability of data

All manuscripts must include a data availability statement. This statement should provide the following information, where applicable:

- Accession codes, unique identifiers, or web links for publicly available datasets
- A description of any restrictions on data availability
- For clinical datasets or third party data, please ensure that the statement adheres to our policy

The raw genotype and phenotype data used to run the association analyses in all separate cohorts are protected and are not available due to data privacy laws. The GWAS meta-analysis summary statistics generated in this study have been deposited in the GWAS Catalog database under accession numbers GCST90565869 [https://ftp.ebi.ac.uk/pub/databases/gwas/summary_statistics/GCST90565001-GCST90566000/GCST90565869/], GCST90565870 [https://ftp.ebi.ac.uk/pub/databases/gwas/summary_statistics/GCST90565001-GCST90566000/GCST90565870/], GCST90565871 [https://ftp.ebi.ac.uk/pub/databases/gwas/summary_statistics/GCST90565001-GCST90566000/GCST90565871/], and GCST90565872 [https://ftp.ebi.ac.uk/pub/databases/gwas/summary_statistics/GCST90565001-GCST90566000/GCST90565872/]. The source data for figures in the main text are available from Github (https://github.com/jodithea/Sex_differences_genetics_depression), which has been archived on Zenodo and assigned a DOI: 10.5281/zenodo.15233098 [https://doi.org/10.5281/zenodo.15233098] [100].

Research involving human participants, their data, or biological material

Policy information about studies with human participants or human data. See also policy information about sex, gender (identity/presentation), and sexual orientation and race, ethnicity and racism.

Reporting on sex and gender

Sex was considered in the study with both sex-stratified and genotype-by-sex interaction analyses carried out. Sex was defined based on chromosomal composition (XX = female, XY = male) determined from genotype data

Reporting on race, ethnicity, or other socially relevant groupings

All analyses were done on individuals of European ancestry only due to data availability. European ancestry was determined using genotype data.

Population characteristics

Characteristics of the cohorts included in this meta-analysis are described in:

Australian Genetics of Depression Study (AGDS) + QSkin Sun and Health Study (QSkin):
Byrne EM, et al., Cohort profile: the Australian genetics of depression study. BMJ Open, 2020. 10(5):e032580.
Olsen CM, et al., Cohort profile: The QSkin Sun and Health Study. Int J Epidemiol, 2012. 41(4):929-929i.

The BIObanks Netherlands Internet Collaboration (BIONIC):
Huider F, et al., Genomics Research of Lifetime Depression in the Netherlands: The BIObanks Netherlands Internet Collaboration (BIONIC) Project. Twin Research and Human Genetics, 2024. 27(1):1-11.

GLAD+: The GLAD+ Study combines two United Kingdom (UK) cohorts: the Genetic Links to Anxiety and Depression (GLAD) Study (www.gladstudy.org.uk), and the National Institute for Health and Care Research (NIHR) BioResource COVID-19 Psychiatry and Neurological Genetics (COPING) Study.
Davies MR, et al., The Genetic Links to Anxiety and Depression (GLAD) Study: Online recruitment into the largest recontactable study of depression and anxiety. Behaviour Research and Therapy, 2019. 123:103503.

Generation Scotland:
Smith BH, et al., Cohort Profile: Generation Scotland: Scottish Family Health Study (GS:SFHS). The study, its participants and their potential for genetic research on health and illness. Int J Epidemiol, 2013. 42(3):689-700.

	UK Biobank:<br>Bycroft C, et al., The UK Biobank resource with deep phenotyping and genomic data. Nature, 2018. 562(7726):203-209.<br><br>All Of Us:<br>All of Us Research Program Investigators, The "All of Us" research program. New England Journal of Medicine, 2019. 381 (7):668-676.
Recruitment	Recruitment differed by cohort and was not carried out as part of this study - data was used from cohorts which already have published cohort profiles. A brief summary follows:<br><br>In the Australian Genetics of Depression Study (AGDS) participants were recruited via Australian government prescription records or through a media campaign. AGDS used the QSkin Sun and Health Study (QSkin) as a control cohort; a population-based cohort from Queensland, Australia that was invited to participate via a random draw from the electoral roll. The publicly available UK Biobank cohort recruited from across the United Kingdom. The publically available All Of Us Research Program recruited from across the United States. The BIObanks Netherlands Internet Collaboration (BIONIC) is a consortium of 16 Dutch studies and biobanks with various recruitment strategies. The GLAD+ Study combines two United Kingdom (UK) cohorts: the Genetic Links to Anxiety and Depression (GLAD) Study and the National Institute for Health and Care Research (NIHR) BioResource COVID-19 Psychiatry and Neurological Genetics (COPING) Study. The ongoing GLAD Study recruits participants with depression and/or anxiety. During the COVID-19 pandemic, the GLAD Study research team recontacted GLAD participants and healthy volunteers from other NIHR BioResource (https://bioresource.nihr.ac.uk/) studies to conduct the COPING study. Generation Scotland is a cohort study of 7,000 families recruited from the general population of Scotland.<br><br>Further details of recruitment are detailed in the following studies:<br><br>Australian Genetics of Depression Study (AGDS) + QSkin Sun and Health Study (QSkin):<br>Byrne EM, et al., Cohort profile: the Australian genetics of depression study. BMJ Open, 2020. 10(5):e032580.<br>Olsen CM, et al., Cohort profile: The QSkin Sun and Health Study. Int J Epidemiol, 2012. 41(4):929-929i.<br><br>The BIObanks Netherlands Internet Collaboration (BIONIC):<br>Huider F, et al., Genomics Research of Lifetime Depression in the Netherlands: The BIObanks Netherlands Internet Collaboration (BIONIC) Project. Twin Research and Human Genetics, 2024. 27(1):1-11.<br><br>GLAD+: The GLAD+ Study combines two United Kingdom (UK) cohorts: the Genetic Links to Anxiety and Depression (GLAD) Study (www.gladstudy.org.uk), and the National Institute for Health and Care Research (NIHR) BioResource COVID-19 Psychiatry and Neurological Genetics (COPING) Study.<br>Davies MR, et al., The Genetic Links to Anxiety and Depression (GLAD) Study: Online recruitment into the largest recontactable study of depression and anxiety. Behaviour Research and Therapy, 2019. 123:103503.<br><br>Generation Scotland:<br>Smith BH, et al., Cohort Profile: Generation Scotland: Scottish Family Health Study (GS:SFHS). The study, its participants and their potential for genetic research on health and illness. Int J Epidemiol, 2013. 42(3):689-700.<br><br>UK Biobank:<br>Bycroft C, et al., The UK Biobank resource with deep phenotyping and genomic data. Nature, 2018. 562(7726):203-209.<br><br>All Of Us:<br>All of Us Research Program Investigators, The "All of Us" research program. New England Journal of Medicine, 2019. 381 (7):668-676.
Ethics oversight	All protocols and questionnaires for both the AGDS and QSkin cohorts were approved by the QIMR Berghofer Medical Research Institute Human Research Ethics Committee (P2118, P1309 and P2034).<br>Generation Scotland ethical approval for the original data collection was obtained from the Tayside Committee on Medical Research Ethics A (ref 05/S1401/89). Generation Scotland is currently approved as a Research Tissue Bank by the East of Scotland Research Ethics Service (ref 20/ES/0021).<br>The GLAD Study was approved by the London - Fulham Research Ethics Committee on 21st August 2018 (REC reference: 18/LO/1218) following a full review by the committee. The NIHR BioResource has been approved as a Research Tissue Bank by the East of England Cambridge Central Committee (REC reference: 17/EE/0025).<br>BIONIC: All relevant ethical regulations were followed. As BIONIC is comprised of multiple studies, ethical approval was granted by the respective institutional review boards or ethics committees of each participating study. Full details on the ethical approvals and participating cohorts are described in Huider et al., 2024<br>Huider F, et al., Genomics Research of Lifetime Depression in the Netherlands: The BIObanks Netherlands Internet Collaboration (BIONIC) Project. Twin Research and Human Genetics, 2024. 27(1):1-11.<br>Ethics approval for the UK Biobank study was obtained from the North West Centre for Research Ethics Committee (11/NW/0382).<br>The All of Us Research Program protocol was approved by the All of Us Institutional Review Board, overseen by the United States National Institutes of Health (NIH).

Note that full information on the approval of the study protocol must also be provided in the manuscript.

Field-specific reporting

Please select the one below that is the best fit for your research. If you are not sure, read the appropriate sections before making your selection.

- [x] Life sciences
- [ ] Behavioural & social sciences
- [ ] Ecological, evolutionary & environmental sciences

For a reference copy of the document with all sections, see nature.com/documents/nr-reporting-summary-flat.pdf

Life sciences study design

All studies must disclose on these points even when the disclosure is negative.

Sample size	Sample size was determined by the data available in each of the cohort studies and published GWAS summary statistics included in the meta-analysis.
Data exclusions	Data exclusions were made using standard criteria for GWAS; data missingness, poor quality genotyping and variants with a minor allele frequency below 0.01. Individuals of non-European ancestry were excluded from analysis, using genotype data.<br>These data exclusions were decided before analyses were carried out.
Replication	Replication of the sex-stratified GWAS was carried out using the Generation Scotland cohort. The number of SNPs with a concordant effect size direction was not significantly greater than expected by chance in both females (concordance = 69% [95% CI: 45 - 100%], H0: concordance = 0.5 [p = 0.11]) and males (concordance = 71% [95% CI: 34 - 100%], H0: concordance = 0.5 [p = 0.23]) (Supplementary Data 6-7). However, the replication cohort is small and the 95% confidence intervals are large suggesting the power of this replication is low.
Randomization	Randomisation was not applicable to this GWAS meta-analysis study which is a retrospective study of Major Depressive Disorder cases and controls.
Blinding	Blinding was not applicable to this GWAS meta-analysis study which is a retrospective study of Major Depressive Disorder cases and controls.

Reporting for specific materials, systems and methods

We require information from authors about some types of materials, experimental systems and methods used in many studies. Here, indicate whether each material, system or method listed is relevant to your study. If you are not sure if a list item applies to your research, read the appropriate section before selecting a response.

Materials & experimental systems

n/a	Involved in the study
[x]	[ ] Antibodies
[x]	[ ] Eukaryotic cell lines
[x]	[ ] Palaeontology and archaeology
[x]	[ ] Animals and other organisms
[x]	[ ] Clinical data
[x]	[ ] Dual use research of concern
[x]	[ ] Plants

Methods

n/a	Involved in the study
[x]	[ ] ChIP-seq
[x]	[ ] Flow cytometry
[x]	[ ] MRI-based neuroimaging

Plants

Seed stocks	*Report on the source of all seed stocks or other plant material used. If applicable, state the seed stock centre and catalogue number. If plant specimens were collected from the field, describe the collection location, date and sampling procedures.*
Novel plant genotypes	*Describe the methods by which all novel plant genotypes were produced. This includes those generated by transgenic approaches, gene editing, chemical/radiation-based mutagenesis and hybridization. For transgenic lines, describe the transformation method, the number of independent lines analyzed and the generation upon which experiments were performed. For gene-edited lines, describe the editor used, the endogenous sequence targeted for editing, the targeting guide RNA sequence (if applicable) and how the editor was applied.*
Authentication	*Describe any authentication procedures for each seed stock used or novel genotype generated. Describe any experiments used to assess the effect of a mutation and, where applicable, how potential secondary effects (e.g. second site T-DNA insertions, mosiacism, off-target gene editing) were examined.*
